# *Chlamydia* spp. development is differentially altered by treatment with the LpxC inhibitor LPC-011

**DOI:** 10.1186/s12866-017-0992-8

**Published:** 2017-04-24

**Authors:** Erik D. Cram, Daniel D. Rockey, Brian P. Dolan

**Affiliations:** Department of Biomedical Sciences, College of Veterinary Medicine, Oregon State University, 105 Magruder Hall, Corvallis, OR 97331 USA

## Abstract

**Background:**

*Chlamydia* species are obligate intracellular bacteria that infect a broad range of mammalian hosts. Members of related genera are pathogens of a variety of vertebrate and invertebrate species. Despite the diversity of *Chlamydia*, all species contain an outer membrane lipooligosaccharide (LOS) that is comprised of a genus-conserved, and genus-defining, trisaccharide 3-deoxy-D-manno-oct-2-ulosonic acid Kdo region. Recent studies with lipopolysaccharide inhibitors demonstrate that LOS is important for the *C. trachomatis* developmental cycle during RB- > EB differentiation. Here, we explore the effects of one of these inhibitors, LPC-011, on the developmental cycle of five chlamydial species*.*

**Results:**

Sensitivity to the drug varied in some of the species and was conserved between others. We observed that inhibition of LOS biosynthesis in some chlamydial species induced formation of aberrant reticulate bodies, while in other species, no change was observed to the reticulate body. However, loss of LOS production prevented completion of the chlamydial reproductive cycle in all species tested. In previous studies we found that *C. trachomatis* and *C. caviae* infection enhances MHC class I antigen presentation of a model self-peptide. We find that treatment with LPC-011 prevents enhanced host-peptide presentation induced by infection with all chlamydial-species tested.

**Conclusions:**

The data demonstrate that LOS synthesis is necessary for production of infectious progeny and inhibition of LOS synthesis induces aberrancy in certain chlamydial species, which has important implications for the use of LOS synthesis inhibitors as potential antibiotics.

## Background

Members of the genus *Chlamydia* are obligate intracellular, intravacuolar, bacteria that can establish persistent infections in a variety of host species. The most clinically prominent species in humans is *C. trachomatis,* which causes serious diseases including pelvic inflammatory disease, hydrosalpinx, and infertility in the female genital tract or trachoma in the eye [1, 2]. Chlamydia pneumoniae infection is very common in humans and leads to respiratory disease. Veterinary chlamydial pathogens include *C. caviae, C. muridarum, C. abortus,* and *C. suis* which infect guinea pigs, mice, sheep, and pigs respectively [3–10]. Infections of other species by chlamydiae are increasingly being identified [11, 12].

Although there are a wide variety of hosts and diseases associated with chlamydial infection, there are many common aspects of basic chlamydial biology. All *Chlamydia* spp. undergo a biphasic developmental cycle inside host cells. Infectious, metabolically inert elementary bodies (EBs) attach and enter the host cell and differentiate, forming metabolically active reticulate bodies (RBs). Following several rounds of binary fission, RBs then re-differentiate back to EBs, in preparation for release and a second round of infection. After the inclusion reaches maturity, bacteria are released from the host cell by either lysis or extrusion continuing the cycle of infection [13]. While this process represents the typical, unobstructed chlamydial developmental cycle, encountering stress factors such as nutrient starvation, host interferon-γ (IFNγ), coinfection with herpesvirus, and exposure to antibiotics causes RBs to become aberrant [14–20]. Upon absence of the stressor, the aberrant state of RBs is reversible resulting in continued production of infectious progeny.

There are also many structural components that are similar among these organisms. All *Chlamydia* species have a common cell wall/outer membrane structure that includes a highly conserved lipooligosaccharide (LOS) molecule with a trisaccharide Kdo region in the order α-Kdo-(2 → 8)-α-Kdo-(2 → 4)-α-Kdo [21]. LOS has multiple functions including the generation of infectious EBs and facilitating attachment and entry of EBs into the host cell [22, 23]. It is unclear if these properties are consistent across species, or if there are novel roles for LOS yet to be discovered.

Host CD8^+^ cytotoxic T cells are responsible for eliminating self-cells that have become infected with intracellular pathogens. Several recent reports have suggested that *C. trachomatis* can evade CD8^+^ T cell recognition using multiple mechanisms, such as up-regulating the negative T cell regulating ligand PD-L1 [24], preventing expression of perforin in CD8^+^ T cells [25], and enhancing host-peptide presentation to perhaps prevent chlamydial-peptide presentation [26]. Understanding how bacterial infection alters host-immune responses is therefore important for both treatment and vaccine development.

Here we utilize LPC-011 (LPC), a potent inhibitor of LpxC in the chlamydial LOS biosynthesis pathway [22], to examine the sensitivity and growth phenotype on other species of *Chlamydia*. We observed that growth in the presence of LPC at the minimum effective concentration (MEC) produced either normal or aberrant RBs in a species-specific manner. This finding suggests a role of LOS in active RB metabolism as well as RB- > EB differentiation. Furthermore, we find that inhibition of LOS synthesis prevents enhanced host-peptide presentation of defective ribosomal products (DRiPs) substrates [27, 28]. We conclude that inhibition of LOS synthesis has differential species-effects on the *Chlamydia* RB formation but alters host-peptide presentation in all species tested.

## Methods

### Cell lines, reagents, and organisms

Cultured murine fibroblast McCoy cells (ATCC® CRL-1696™) were grown in DMEM (Life Technologies) with 10% FBS (Life Technologies) at 37 °C in 5% CO_2_. Infections with *Chlamydia trachomatis* L2/pBRmChE (a generous gift from Robert J. Suchland, University of Washington), *Chlamydia caviae* GPIC*, Chlamydia trachomatis* J6276, *Chlamydia abortus* (Oregon placental isolate OP5C), *Chlamydia suis* R19, and *Chlamydia muridarum* were all carried out in McCoy cells as previously described [26]. The human B lymphoblastoid cell line JY, expressing the Shield-1 Controlled Recombinant Antigenic Protein (SCRAP) [26], were grown in RPMI (Life Technologies) supplemented with 7.5% FBS (Life Technologies), GlutaMAX (Gibco, 20 mM), and HEPES (Gibco, 10 mM). Cells were incubated at 37 °C in 6% CO_2_. The synthesis of LPC-011 was carried out in the laboratory of P. Zhou (Duke University) as previously described [29].

### Antibody labeling and fluorescence microscopy

McCoy cells were grown to 20% confluency on glass coverslips within individual wells of a 24-well tissue culture treated plate and infected with the indicated chlamydial strain. After 48 hpi (unless stated otherwise) medium was removed and cells were fixed with 100% methanol for ten minutes at room temperature. After removal of methanol, cells were washed 3X with Dulbecco’s phosphate-buffered saline (DPBS: Life Technologies). LOS was labeled with mAb EVI-HI ([30] a gift from Harlan Caldwell), Hsp60 was labeled with mAb B9, *C. trachomatis* inclusion membrane protein A (IncA) with mAb 12 E7, and *C. cavaie* IncA with mAb 17 [31–34]. After an hour of incubation and 3X washes with DPBS, secondary labeling was performed with goat-anti-mouse IgG1 or IgG2a, conjugated, respectively, to FITC (fluorescein) or TRITC (tetramethylrhodamine) (Southen Biotech). VectaShield (Vector Laboratories) containing 4′,6-diamidino-2-phenylindole DAPI (Sigma-Aldrich) was used to stain DNA. Images were collected with a Leica DML scope fitted with a Retiga 2000R camera and processed with QCapture Pro 6.0 software (Q Imaging). To determine the MEC of LPC for the production of LOS in each chlamydial species, cells were treated with 10-fold serially diluted LPC and visualized for LOS production by fluorescence microscopy. Once a potential range for LPC was determined, 2-fold serial dilutions of LPC were conducted to determine a final MEC.

### Chlamydial growth kinetics and quantification

McCoy cell monolayers in 24 well trays were infected with chlamydiae at an MOI of 0.5 either in the presence of LPC at indicated concentrations or in the presence of the DMSO diluent. At each indicated time point, all media was removed and cells were rinsed with DPBS. EBs were released from cells by incubation in sterile water for 10 min, disrupted with serial pipetting, and stored at −80 °C. Samples were thawed and then processed using the DNeasy Blood and Tissue kit (Qiagen) with the single modification of adding dithiothreitol (DTT; 5 mM) before incubating samples with proteinase K at 56 °C for 1 h. Genome copy number was determined by TaqMan q-PCR using probes to *ompA* (*C. caviae)* or *hsp60* (L2) with plasmids containing *ompA* or *hsp60* for standards as previously described [35, 36].

### Growth recovery assay

McCoy cells were grown to a fully confluent monolayer in a 24-well tissue culture tray and were infected with either *C. caviae* or *C. trachomatis* L2/pBRmChE at an MOI of 0.5. At *T* = 0, cells were cultured in triplicate in DMEM supplemented with 10% FBS, 1 μg/mL cycloheximide (Sigma-Aldrich) plus or minus added LPC. The concentration of LPC is indicated for each experiment in the results. At 24 hpi, medium was removed and cells were washed 3X with DPBS and incubated in fresh DMEM +10% FBS and 1 μg/mL cycloheximide. At 48 hpi, cells were lysed in H_2_O and an equal volume of 2X PBS was then added back to cells which were then removed and pooled in a single 2 mL tube prior to storage at −80 °C. A new monolayer of McCoy cells in wells of a 24-well tissue culture tray was then infected in triplicate with EB-containing lysed cell inoculum in a dilution series. At 48 hpi, cells were methanol-fixed and stained with antibody to Hsp-60. The number of inclusions in each of ten 40X fields were counted and a mean value calculated. The averages were multiplied by the number of total possible fields in a well (1019) and then divided by the dilution factor to determine IFU/mL.

### Phylogenetic analysis of LpxC

The LpxC sequence from *C. caviae* (NCBI Reference Sequence: WP_041462191.1), *C. suis* (NCBI Reference Sequence: WP_035406881.1), *C. muridarum* (NCBI Reference Sequence: WP_010231676.1), *C. trachomatis* J/6276 (GenBank: AGS02347.1), *C. trachomatis* L2 434/Bu (GenBank: CAP04233.1), *C. abortus* (NCBI Reference Sequence: WP_041461302.1), and *Escherichia coli* K12 (GenBank: BAB96664.1) were aligned by ClustalW and phylogenic comparisons made using MegAlign in Laser Gene.

### Infection and antigen presentation assay

Infection of JY SCRAP cells with *Chlamydia* and quantitation of antigen presentation was carried out as previously described [26] with minor modifications. The difference was that cells infected with *C. muridarum*, were harvested at 20 hpi because *C. muridarum* replicates at a faster rate than other chlamydial species tested. All cells were treated with Shield-1 for 12 h before analysis. Cells were stained with the monoclonal antibody RL15A, which specifically recognizes the SVGGVFTSV (hereafter SVG) peptide bound to HLA-A2 molecules. Labeled cells were stained in triplicate and the average mean fluorescence intensity (MFI) of the population was acquired by flow cytometry on an Accuri C6 instrument. The average MFI of the technical replicates is shown and Student’s t-tests were performed using Graphpad Prism 6 software. Statistical significance was determined with alpha values of 0.05.

## Results

### *C. trachomatis* L2 is less sensitive to the LOS biosynthesis inhibitor LPC than *C. caviae*

**Fig. 1.**
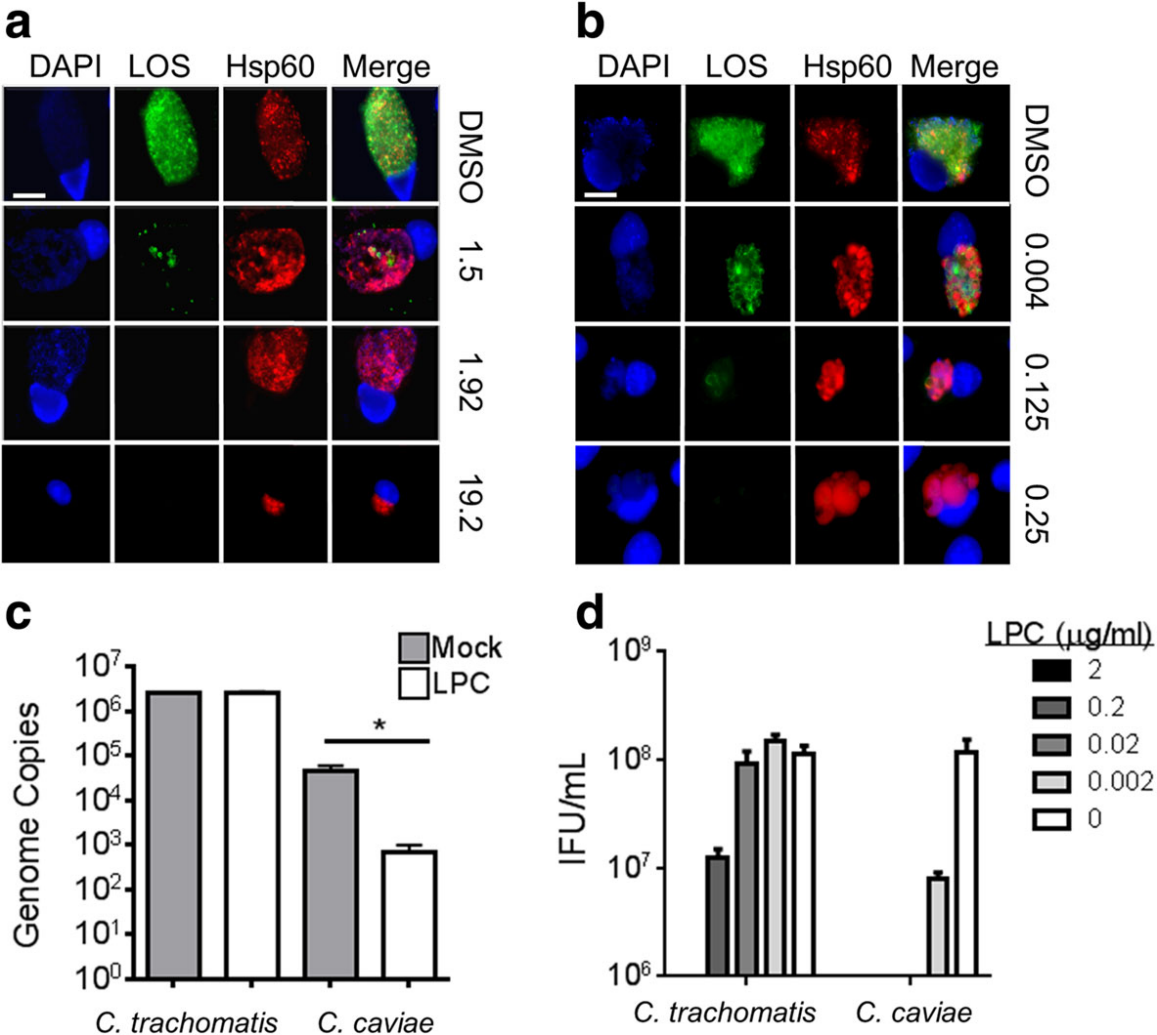
Treatment of *C. trachomatis* and *C. cavaie*-infected cells with LPC reveals an aberrant phenotype and differential sensitivity of each species to LPC**. a** *C. trachomatis*-infected McCoy cells were treated with either DMSO, or the indicated concentrations of LPC (in μg/ml) for 48 h at which time LOS and Hsp60 were visualized by confocal microscopy. The MEC was calculated to be 1.92 μg/ml. **b** Same as in (**a**) except cells were infected with *C. caviae* and treated with lower concentrations of LPC. The MEC for *C*. *caviae* is 0.25 μg/ml. Treatment of LPC on C. caviae infected cells resulted in the formation of aberrant RBs both at, and well below the MEC of drug. **c** *C. trachomatis* L2 and *C. caviae* infected cells were treated with DMSO or the appropriate MEC of LPC and cells were harvested 48 h.p.i. The number of chlamydial-genome copies was calculated by qPCR. Treatment of *C. trachomatis* L2 with 1.92 LPC μg/mL (MEC) did not result in a reduction of genome copy number compared to mock-treated L2, while treatment of C. caviae with 0.25 μg/mL resulted in 2-log decrease of genome copy number (*, *P* < 0.05). **d** EBs from LPC-treated cells infected with either *C. trachomatis* or *C. caviae* were determined. Scale bar is 10 μm

We used a serial dilution approach to determine the concentration of LPC required to completely inhibit *C. trachomatis *L2 LOS biosynthesis in McCoy fibroblast cells. At 48 hpi, cells were fixed with methanol, stained for LOS and Hsp60 and visualized with fluorescence microscopy. In agreement with Nguyen et al. [22] the MEC was determined to be 1.92 μg/mL, as LOS production was still detected when cells were treated with 1.5 μg/mL LPC but not 1.92 μg/mL (Fig. 1a). Unlike *C. trachomatis,* a much lower dose of LPC (0.25 μg/mL) was needed to inhibit LOS accumulation in *C. caviae* infected cells (Fig. 1b). Perhaps more striking than a lower MEC was the observation that the chlamydial inclusion structure was visibly altered at sub-MEC doses of LPC in *C. caviae*, but not *C. trachomatis*-infected cells. Aberrant reticulate bodies (ABs) were indeed induced in *C. trachomatis*-infected cells, but required a 10 fold increase in LPC, relative to the MEC however, treatment beyond 2.0 μg/mL appeared to stress host cells and may be possibly toxic as has been previously reported (Fig. 1a). In contrast, ABs in *C. caviae*-infected cells were visible at 0.004 μg/mL LPC, a concentration far below the LPC MEC in this species (Fig. 1b). We also examined replication of each species when cells were treated with LPC at their respective MECs. LPC treatment of *C. trachomatis* infected cells did not alter chlamydial genome replication, while treatment of LPC on *C. caviae* resulted in nearly a 2-log genome copy reduction by 36 hpi (Fig. 1c, *P* < 0.05).

We then examined the ability of infected cells to produce infectious EBs when treated with different concentrations of LPC. Production of *C. trachomatis* EBs was reduced 10 fold at a sub-MEC dose of LPC, and no EBs were detected when treated at the MEC in agreement with Nguyen et al. [22]. *C. caviae* EB production was reduced at LPC concentrations ~100-fold lower than the MEC for LOS production and no EBs were detected when LPC concentrations exceeded 0.002 μg/ml. These data demonstrate that two species of *Chlamydia* have differential sensitivity to the inhibition of LOS synthesis, which also manifests differently with regards to the production of ABs, genome replication, and EB production in treated, infected cells.

### Sensitivity of other chlamydial species to LPC

**Fig. 2.**
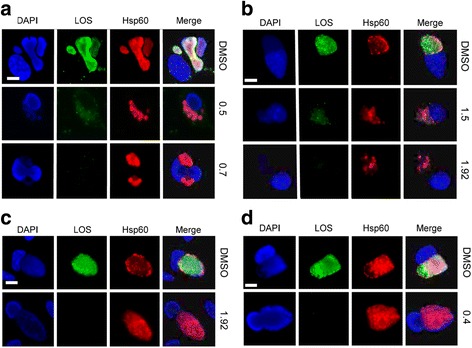
*Chlamydia* spp. inclusion phenotype and sensitivity to LPC. McCoy cells were infected with *C. abortus* (**a**), *C. suis* (**b**), *C. trachomatis* serovar J (**c**), or *C. muridarum* (**d**) and treated with either DMSO or LPC at the indicated concentrations (in μg/mL). At 48 hpi, cells were stained with DAPI or antibodies to LOS or Hsp60 and visualized by fluorescent microscopy. Scale bar is 10 μm

*C. caviae* and *C. trachomatis* are both members of the family Chlamydiaceae but are both distantly related and grouped in two different phylogenetic clades, formerly classified into the genera *Chlamydophila* and *Chlamydia* [37, 38]. In order to determine if the aberrant outcome of LPC treatment is distinguishable by clades, *Chlamydia abortus and C. caviae* (formerly genus *Chlamydophila*), and members of the second clade *C. trachomatis* J6276, *C. suis* and *C. muridarum* were treated with LPC and MEC, and aberrant RB formation monitored. *C. abortus* exhibited greater tolerance to LPC than *C. caviae* with an MEC of 0.7 μg/mL (Fig. 2a), however, the *C. abortus* inclusion phenotype was represented by few, large ABs even at LPC levels permissive of LOS biosynthesis (Fig. 2a). The MEC for LPC in *C. suis* was 1.92 μg/mL with LOS detectable at 1.5 μg/mL, however, similar to *C. abortus* and *C. caviae*, but unlike *C. trachomatis* L2, few, large ABs were present at both concentrations (Fig. 2b). *C. trachomatis* J6276 treated with 1.92 μg/mL LPC had identical inclusion phenotypes to *C. trachomatis* L2, which is manifested as inclusions appearing similar to their vehicle-treated controls (Fig. 2c). *C. muridarum* developed full-sized typical inclusions in the presence of LPC. However, unlike *C. trachomatis* serovars, *C. muridarum* was far more sensitive to LPC (MEC of 0.4 μg/mL, Fig. 2d).

**Fig. 3.**
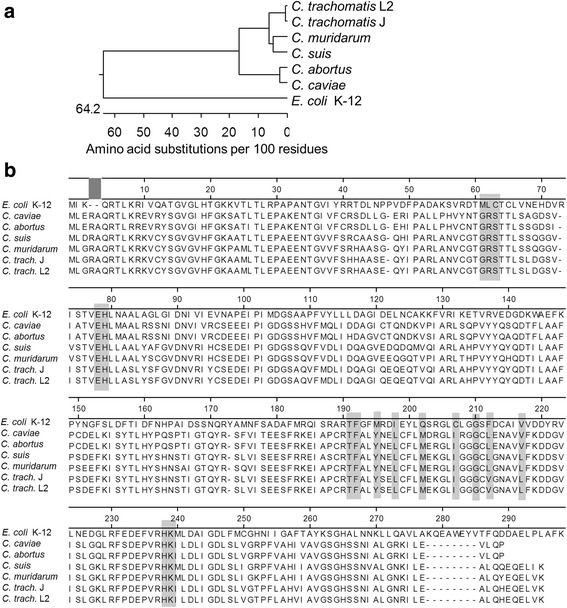
Amino acid composition of LpxC does not explain differential sensitivity to LPC-011. **a** Phylogenic-tree analysis of the LpxC enzyme in the *Chlamydia* species used in this study aligned to *E. coli* LpxC. **b** Clustal-W Alignment of the first 300 amino acids of LpxC. Numbering refers to amino acid position in the *E. coli* LpxC. Residues that are likely involved in LPC interactions are shaded

The differences in sensitivity to LPC and phenotypic outcome may be the result of dissimilar LpxC function in different species, that may not be reflective of the phylogenetic relationship based on 16S ribosomal RNA sequences. To address this, LpxC amino acid sequences from *C. muridarum, C. trachomatis* L2 434/Bu, *C. trachomatis* J6276, *C. abortus*, *C. caviae, C. suis,* and *E. coli* K12 were aligned. The phylogenic tree based upon LpxC alignment is similar to previously published 16S phylogenetic analysis (Fig. 3a) [38]. Therefore the differences between chlamydial species sensitivity to the loss of LOS synthesis is not a function of simple phylogenetic distance from one another. Simple phylogeny alone may not explain differential sensitivity to LPC-011 treatment. We therefore compared the amino acid sequences of LpxC directly (Fig. 3b). Previous crystal structures of bacterial LpxC protein complexed with LPC-011 or other similar compounds have been described [29, 39, 40] and residues that are likely to be important for interacting with LPC are shaded (Fig. 3b). In some instances, such as M61, L62, C63, M195, and I198, chlamydial species are completely divergent from *E. coli*, but all chlamydial species share the same residue as each other. The only amino acid residues which differ between chlamydial species and are likely to be involved in LPC interactions are in the hydrophobic residues that interact with the phenyl ring of LPC. F212 in *E.coli* has changed to leucine in all species of chlamydia except *C. trachomatis* where it is valine. Similarly, *E. coli* V217 is valine in *C. caviae* and *C. abortus* but has mutated to leucine in the remaining chlamydial species. Therefore, differential sensitivity to LPC-011 cannot be explained by the amino acid sequences of LpxC.

### *C. caviae* multi-lobed inclusion phenotype is altered by treatment with LPC

**Fig. 4.**
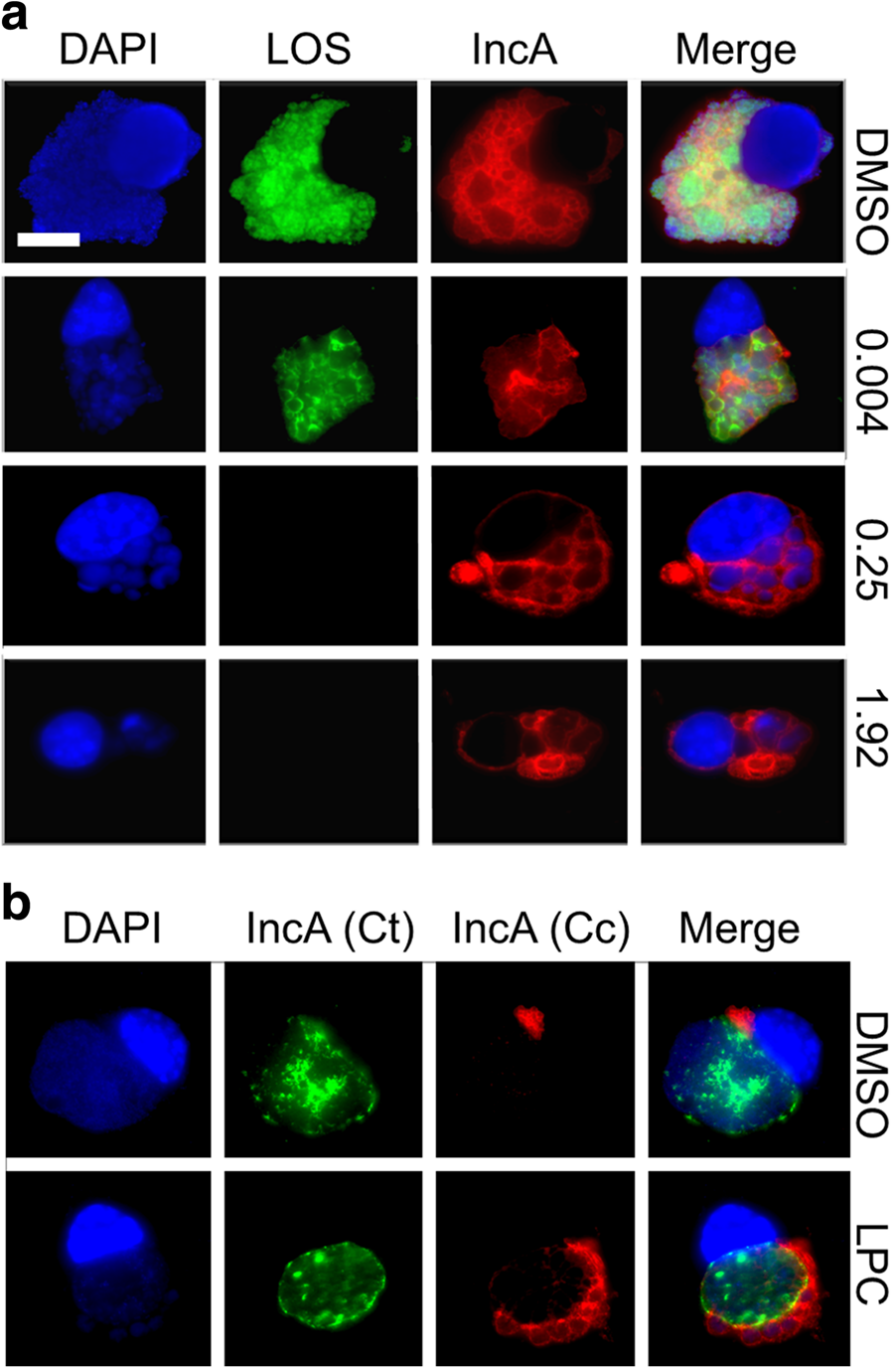
Treatment with LPC alters the multi-lobed phenotype of *C. caviae* infection. **a** Increasing concentrations of LPC (in μg/mL) results in diminished *C. caviae* inclusion compartments and few, large ABs as visualized by DAPI. **b** Treatment of LPC (1.92 μg/mL) on cells dually infected with *C. trachomatis* L2 (Ct) and *C. caviae* (Cc) did not allow for the formation of mixed-species inclusions. Scale bar is 10 μm

Unlike *C. trachomatis,* inclusion division and septation are intimate biological process during the *C. caviae* developmental cycle [41]. In order to determine if the inhibition of LOS biosynthesis affects the multi-lobed inclusion phenotype, *C. caviae-*infected McCoy cells were treated with increasing doses of LPC, and then fixed cells were examined with antibodies to the inclusion membrane protein A (IncA) using fluorescence microscopy. LPC treatment, at doses both below and above the MEC, resulted in fewer inclusion compartments with few, large ABs (Fig. 4a) suggesting LOS as an essential component of *C. caviae* development. Inclusion bodies of *C. trachomatis* L2 and *C. caviae* do not fuse when co-infecting the same cell [42]. To determine if alteration of the inclusion and absence of LOS by LPC treatment would lead to a fusion event, we examined co-infected cells treated with 1.92 μg/ml LPC. No fused inclusions were noted (Fig. 4b) indicating that inclusion development remains segregated in the absence of LOS.

### *C. trachomatis* L2 and *C. caviae* aberrant body formation are inversely sensitive to ampicillin and LPC

**Fig. 5.**
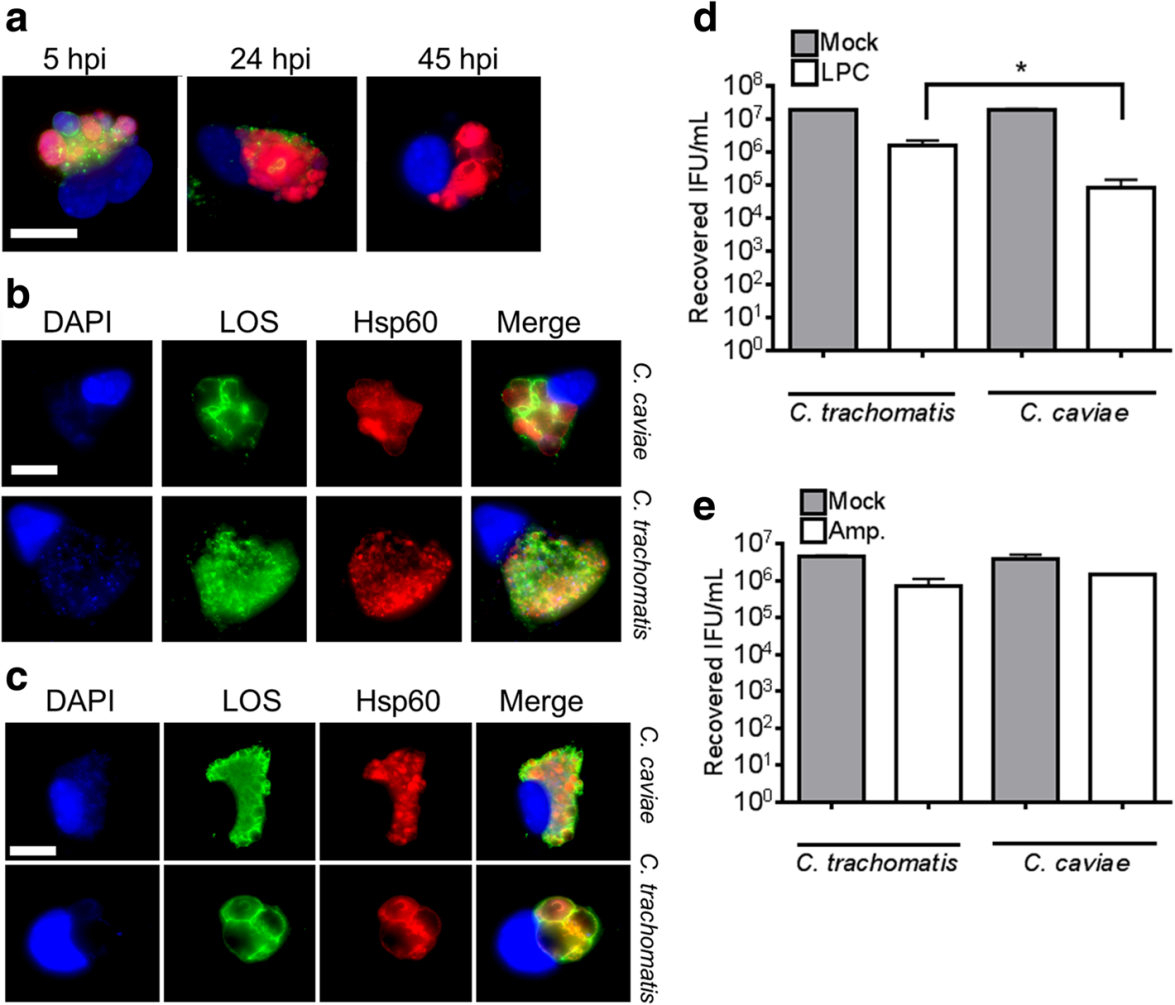
*C. caviae* and *C. trachomatis* are inversely sensitive to ampicillin and LPC. **a** *C. caviae* infected cells were treated with 0.25 μg/mL LPC at 0 hpi and at 5, 24, and 45 hpi cells were washed and drug was removed. At 48 hpi, infected cells were methanol fixed, and labeled with the anti-LOS mAb (green) and anti-Hsp60 mAb (red) and total DNA with DAPI *(blue*). At 24 hpi, a modest production of LOS was observed within *C. caviae* inclusions. **b** *C. caviae* and *C. trachomatis* L2-infected cells were treated with LPC at their respective MECs for 24 hpi, and allowed to recover for an additional 24 h in the absence of drug. Cells were then labeled and visualized as in (**a**). **c** Cells were treated with ampicillin in place of LPC. **d** and **e** Infected cells were treated with LPC (**d**) or ampicillin (**e**) and allowed to recover as above. Infectious progeny titers were determined by re-infecting McCoy cells (*, *P* < 0.05). Scale bar is 10 μm

During the persistent state of chlamydial infections smaller inclusions containing lesser numbers of abnormally large, ABs are routinely detected. Persistence both in vitro and in vivo are induced by treatment with antibiotics such as penicillin, IFNγ production by the host cell, and nutrient abundance [43]. To determine if LPC can induce a persistent phenotype in vitro, we compared the ability of *C. caviae* and *C. trachomatis* L2 RBs to differentiate into infectious EBs following recovery from exposure to LPC. *C. caviae* was exposed to LPC and at 5, 24 and 45 hpi, infected cells were washed three times with PBS and replaced with fresh media until 48 hpi when cells were examined by fluorescence microscopy to determine if LOS production had recommenced. We found 24 h of recovery was enough time to observe modest production of LOS in infected cells (Fig. 5a). We then directly compared *C. trachomatis* L2 and *C. caviae* infected cells, treated with LPC for 24 h and then cultured in the absence of LPC for an additional 24 h (Fig. 5b). *C. trachomatis* L2 infected cells produced typical inclusions following LPC removal while *C. caviae* infected cells had few, large inclusions that differed from typical *C. caviae* inclusions, despite the presence of LOS. The inclusion-formation phenotypes were reversed when infected cells were exposed to ampicillin. *C. trachomatis* L2 infected cells struggled to produce typical inclusions while *C. caviae* infected cells had multiple, lobed inclusions (Fig. 5c) following recovery from drug exposure. In addition to alterations to inclusion morphology, we also determined the effect of LOS recovery on the production of infectious progeny. Following removal of LPC at 24 hpi, and an additional 24 h of culture, *C. caviae* production was reduced by 233 fold, whereas *C. trachomatis* progeny were reduced by 12-fold (Fig. 5d). In contrast, 24 h exposure to ampicillin (10 μg/mL) with a subsequent 24 h recovery yielded a 3-fold reduction in *C. caviae* IFU/mL while *C. trachomatis* L2 IFU/mL was reduced 7-fold (Fig. 5e). Production of *C. trachomatis* EBs following ampicillin removal occurred even though inclusion morphology did not resemble untreated cells, as has been previously noted [44]. Therefore, differential sensitivity to LPC not only alters morphology of the inclusion, but also the ability of different *Chlamydia* to recover from the loss of LOS synthesis.

### Treatment of LPC on *Chlamydia* spp. infection abrogates enhanced MHC Class I self-antigen presentation phenotype

**Fig. 6.**
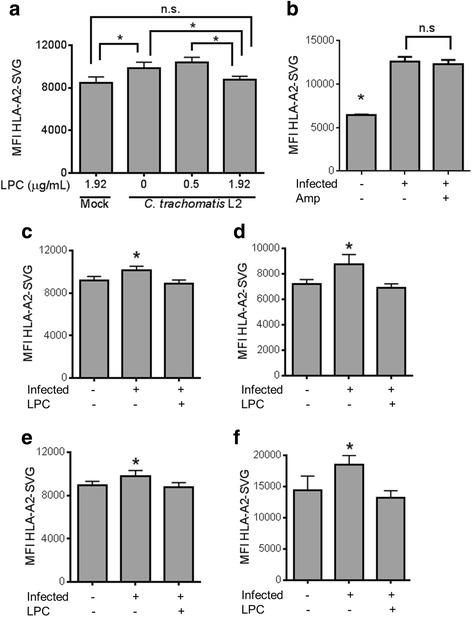
*Chlamydia* enhance self-peptide presentation in a LOS-dependant mechanism: **a**. L2 infected cells were treated with mock, sub-MEC and MEC concentrations of LPC. At the MEC of LPC (1.92 μg/mL) surface HLA-A2-SVG were reduced to mock-infected levels. A lower concentration of 0.5 μg/mL LPC did not rescue increased surface HLA-A2-SVG phenotype. **b**. Similar to (**a**), *C. trachomatis* L2-infected cells were either treated with ampicillin (10 µg/mL) or mock-treated and surface HLA-A2-SVG complexes were measured. **c**. Surface levels of HLA-A2-SVG were increased during *C. caviae* infection which was abrogated by treatment with LPC (2 μg/m). The same results were true for *C. muridarum* (**d**), *C. abortus* (**e**), and *C. suis* (**f**) (*, *P* < 0.05)

The direct MHC class I antigen presentation pathway is responsible for ensuring short peptides from both host and intracellular pathogens are presented to cytotoxic T cells. Using a cell-based model system, we have previously demonstrated that *C. trachomatis* and *C. caviae* infection enhances the presentation of a model host-peptide derived from the DRiP form of the antigen [26]. Furthermore, treatment of *C. trachomatis*-infected cells with LPC reversed the antigen presentation phenotype, suggesting that it was mediated by LOS. In order to further show that LOS is important to the enhanced self-antigen presentation phenotype, *C. trachomatis* L2 infected cells were treated with a concentration of LPC that is permissive to LOS biosynthesis and compared to LPC treatment at the MEC (Fig. 6a). Similar to our previous results, treatment at the MEC of LPC rescued host-peptide presentation to levels similar to uninfected cells. However, LPC treatment below the MEC (0.5 μg/ml) resulted in increased host-peptide presentation similar to infected cells treated with vehicle alone. To determine if aborted infection induced by LPC treatment was the cause of enhanced antigen presentation, we treated *C. trachomatis* infected cells with ampicillin, which induces aberrant body formation but still allows for LOS biosynthesis. Ampicillin treatment did not reverse the enhanced antigen presentation phenotype observed upon *C. trachomatis* infection, suggesting that LOS and not aberrancy is responsible for enhancing self-antigen presentation (Fig. 6b). We then sought to establish which, if any, other *Chlamydia* species increase self-antigen presentation upon infection and the necessity of LOS biosynthesis for that phenotype. *C. caviae*-infected cells showed an increase in surface HLA-A2-SVG that was abrogated by treatment with LPC (Fig. 6c). Similarly, *C. muridarum* (Fig. 6d), *C. abortus* (Fig. 6e), and *C. suis* (Fig. 6f) all were able to enhance presentation of host-peptides from DRiP substrates, and the enhanced presentation phenotype was abrogated by treatment of LPC (2 μg/mL). Therefore, synthesis of chlamydial LOS can enhance the presentation of peptides from host DRiPs.

## Discussion

The reason for the highly conserved nature of the chlamydial LOS molecule between chlamydial species remains one of the greatest mysteries in this system [45]. Various selective forces within a range of host organisms have not yielded much change in the structure within the genus. Known interactions of LOS with host biology include cell attachment and entry, as well as interacting as a hemagglutinin with erythrocytes [23, 46, 47]. Little is known about the role of LOS in the chlamydial developmental cycle and uncovering this information may be the key to unlocking the anomaly of conserved LOS structure.

In this manuscript, we were able to demonstrate that LOS biosynthesis is important for the successful completion of the bacteria’s development cycle, though there were some striking differences between the different species of *Chlamydia* tested. One of the more striking differences relates to the MEC of LPC for each species, as defined by the dose needed to inhibit LOS production. Synthesis of LOS in the *C. trachomatis* serovars as well as *C. suis* was inhibited at 1.92 μg/mL LPC, similar to previously reported values for *C. trachomatis* [22]. However, the MEC for *C. muridarum*, which is in the same phylogenetic clade as *C. trachomatis* and *C. suis* was 0.4 μg/ml. This sensitivity value was closer to the MEC for the more distally related *C. abortus* (0.7 μg/mL) and *C. caviae* (0.25 μg/mL). Possible explanations for this observation include: 1.) there is a difference in the ability of LPC to access the developing reticulate body and therefore the effective concentration at the site of the enzyme is different or 2.) there are critical elements of LpxC shared between the species with greater sensitivity to LPC that are not reflective in amino acid sequence differences. LpxC from *C. muridarum* clusters most closely with *C. suis* and then to the *C. trachomatis* serovars (Fig. 3a) and direct amino acid comparisons between the species did not reveal any obvious amino acid similarities shared between *C. muridarum*, *C. abortus*, *and C. caviae*, that were excluded from the other species (Fig. 3b), which suggest that the first hypothesis is more likely to be correct.

In addition to the differences in MEC, the resulting phenotype of LpxC inhibition was different between *Chlamydia* species. Inhibiting LOS biosynthesis with the MEC of LPC did not create aberrant reticulate bodies in *C. trachomatis* infected cells, nor did it result in a reduction in genome copy numbers of progeny bacteria, though no infectious bacteria could be recovered from LPC treated cells. ABs were not detected in *C. muridarum*-infected cells, but could be detected in *C. suis*-infected cells, similar to the species in the chlamydophila clade, *C. caviae* and *C. abortus*. This is surprising considering *C. muridarum* was more sensitive to LPC treatment than *C. suis* which had a MEC similar to the *C. trachomatis* serovars. AB formation still occurred when LOS levels were reduced, but not eliminated, and this likely impacts the ability to produce infectious progeny as shown in Fig. 1d. We unfortunately, cannot determine if the loss of EB production is the result of AB formation or loss of LOS. It is likely the two processes are intimately linked in a species-specific manner and therefore determining an exact mechanism is difficult. The formation of functional reticulate bodies is therefore likely to be dependent on the concentration of LOS, with *C. trachomatis* and *C. muridarum* being able to create reticulate bodies with levels of LOS below our detection limit whereas other chlamydial species need slightly higher levels of LOS in order to avoid entering aberrancy.

Many factors are known to induce the persistent state of chlamydia in vitro including amino acid starvation, IFN-γ, NO, β-lactam antibiotics, and now, inhibition of LOS biosynthesis [43, 48, 49]. We directly compared the ability of *C. trachomatis* and *C. caviae* to recover from a persistent state induced by either ampicillin or LPC treatment. Even after removal of LPC, bacterial replication was greatly reduced (12-fold for *C. trachomatis* and 233-fold for *C. caviae*) and ABs were still present in *C. caviae*-infected cells. Bacterial replication was still reduced following recovery from ampicillin treatment (7-fold for *C. trachomatis* and 3-fold for *C. caviae*) but not to the same extent as LPC treatment. Recovery after ampicillin treatment allowed for the formation of multi-lobed inclusions in *C. caviae*-infected cells, though ABs were still present in *C. trachomatis*-infected cells. Despite these ABs, infectious progeny were still produced. It is therefore tempting to speculate that LPC treatment may prove to be a better long-term treatment option for controlling *Chlamydia* infections. As more compounds become available to prevent bacterial growth, it will be important to examine how they can alter *Chlamydia* spp. persistence.

We have previously observed that *C. trachomatis* and *C. caviae* infection can increase the ability of host cells to present self-antigens via the MHC class I antigen presentation pathway [26]. Here we extend these findings to demonstrate that other *Chlamydia* generate a similar phenotype during infection. Cytotoxic T cells can be activated by very low numbers of peptide-MHC complexes at the cell surface [50–52], and it is therefore unknown what the modest increase in antigen presentation observed with a single model antigen means for T cell recognition. However, increased self-peptide-MHC density has been linked to enhanced T cell activation [53], suggesting that enhancing self-antigen presentation may aid in pathogen clearance. Alternatively, the increased numbers of self-peptides presented may lead to increased recognition of cells by self-reactive T cells. Until a complete study of peptidome alterations upon infection is conducted, we can only speculate at the possible outcomes.

We find that inhibition of LOS synthesis reverses the enhanced host-antigen presentation phenotype in all chlamydial species tested, and this does not appear to be the result of induced aberrancy as ampicillin treatment had no effect on antigen presentation but did induce aberrancy. Additionally, decreasing LPC concentrations to levels below the MEC for LOS synthesis did not alter *Chlamydia*-induced antigen presentation, suggesting that LOS is mediating the effect. How LOS exerts a function on MHC class I antigen presentation is unknown. LOS itself could be recognized by intracellular innate immune sensors, in a manner similar to inflammatory caspases which recognizes *E. coli* LPS [54]. Alternatively, loss of LOS may alter the bacterial membranes and prevent functioning secretion systems, which would inhibit bacterial components from entering the host-cell cytosol. If there is a specific chlamydial protein which acts to enhance self-peptide presentation, LPC treatment may simply prevent said factor from reaching its intended target.

## Conclusions

*Chlamydia* spp were sensitive to the inhibition of LOS synthesis by treatment with the LpxC inhibitor LPC-011, however the dose of LPC and the inclusion phenotype differed between species.  Aberrant bodies developed in some species tested resulting in lower bacterial replication, while other species developed inclusions containing morphologically typical RBs.  For all species tested, our data demonstrate that LOS biosynthesis is important both for generation of infectious progeny and necessary for the enhanced surface HLA-A2-SVG phenotype.

## Abbreviations

AB: Aberrant reticulate body
DRiP: Defective Ribosomal Product
EB: Elementary body
HLA: Human Leukocyte Antigen
IFNγ: Interferon-γ
IFU: Infectious units
IncA: Inclusion membrane protein A
LOS: Lipooligosaccharide
LPC: LPC-011
mAb: Monoclonal antibody
MEC: Minimum Effective Concentration
MHC: Major Histocompatibility Complex
RB: Reticulate body
